# The Orthopedic Upper Extremity Surgeon’s Role in the Diagnosis and Treatment of Osteoporosis: Making Use of Opportunistic Imaging

**DOI:** 10.3390/jcm13175095

**Published:** 2024-08-28

**Authors:** Caitlin M. Ward, Eric J. Gullborg, Xavier C. Simcock

**Affiliations:** Department of Orthopaedic Surgery, Rush University Medical Center, Chicago, IL 60612, USA; caitlin_m_ward@rush.edu (C.M.W.); xavier.simcock@rushortho.com (X.C.S.)

**Keywords:** osteoporosis, fragility fracture, opportunistic imaging, upper extremity surgeons, bone mineral density, Hounsfield units, distal radius

## Abstract

Osteoporosis is an underdiagnosed and undertreated public health issue that contributes to a high financial burden on the healthcare system and imposes significant morbidity and mortality on the patient population. Upper extremity orthopedic surgeons are in a unique position to diagnose osteoporosis prior to patients suffering a fragility fracture by using imaging that they already obtain in their current workflow. The use of X-rays and CT scans can effectively diagnose osteoporosis with high sensitivity and specificity. By incorporating these diagnostic methods into standard practice, upper extremity orthopedic surgeons can play a critical role in the early diagnosis and treatment of osteoporosis. This can prevent severe fractures, improve patient outcomes, and reduce the overall healthcare burden by initiating timely treatment and patient education. This review emphasizes the importance of opportunistic imaging in enhancing osteoporosis management and suggests that upper extremity surgeons can significantly and effectively contribute to primary and secondary fracture prevention.

## 1. Introduction

Osteoporosis is the most common bone disease in the United States and heavily contributes to both the financial burden on our healthcare system as well as to the morbidity and mortality of our patient population [1]. Currently, osteoporosis is defined by the World Health Organization (WHO) as having a bone mineral density (BMD) that is measured via Dual Energy X-ray Absorptiometry (DEXA) at a t-score of −2.5 and below, as compared to what is considered to be peak bone mass in a patient population of healthy young female controls. Other diagnostic criteria include a history of fragility fractures, a calculated future overall risk of fracture of 20%, or a specific risk of hip fracture of 3% (WHO). The prevalence in the United States among adults aged 50 and older is 12.6%, 19.6% in women and 4.4% in men. Additionally, the prevalence in women has been found to be increasing, even over the course of the past decade [1]. This healthcare crisis is not limited to the United States, and the worldwide prevalence of osteoporosis is even more pronounced at 18.3–23.1% female and 11.7% male [2].

The most important and costly sequela of osteoporosis is fracture, which has a significant impact on patients’ quality of life and even life expectancy, depending on the fracture’s location. Worldwide, one in three women and one in five men over the age of 50 will experience an osteoporosis-associated fracture in their lifetime, and as the prevalence of osteoporosis grows, so will the number of fractures [3]. It is estimated that by 2050 the global number of hip fractures, one of the most morbid fractures associated with osteoporosis, will increase significantly due to the ageing population of the world, further escalating the healthcare burden [4].

In the USA, over 1.5 million fractures annually could be attributed to osteoporosis [5]. This translates to approximately USD 12–18 billion [5], making primary and secondary prevention of osteoporotic fractures a top priority to help reduce healthcare costs. In response to this public health crisis, programs such as Own the Bone and fracture liaison hospital services (FLS) have been developed and implemented [6]. These programs are helping improve rates of diagnosis and treatment of osteoporosis, but there is still much work to be done.

Orthopedic surgeons are poised as one of the first providers a patient sees once they have experienced a fragility fracture, and they are therefore key to the diagnosis, treatment, and prevention of osteoporosis and its sequelae. However, the orthopedic community has not yet seized this opportunity effectively. Hernlund et al. suggest that potentially 80% of high-risk individuals who have already experienced at least one osteoporotic fracture are not correctly identified and being treated for subsequent osteoporosis [7]. These patients are considered high-risk, as having a history of previous fracture increases the risk of another fracture by 86% [8], therefore, it is imperative to correctly identify and treat them appropriately after the first fracture, if not before. Hip fractures carry the most morbid consequences, with studies indicating 1-year mortality rates of up to 20–24% [9,10], and yet 50% of hip fracture patients have already sustained at least one prior fracture, thus indicating a missed chance to intervene prior to hip fracture [11].

Working within a multi-disciplinary team that includes primary care providers, geriatricians, and osteoporosis specialists/endocrinologists, orthopedic surgeons have a responsibility to help address this growing bone health epidemic. Upper extremity surgeons in particular play a key role, as distal radius and proximal humerus fractures make up two of the three most common fractures seen in the elderly population [12]. Patients who have suffered a distal radius fracture have been shown to be at five-times higher risk than their counterparts of sustaining a hip fracture within one year [12]. By treating distal radius fractures as sentinel events, hand and upper extremity surgeons can improve rates of osteoporosis diagnosis and treatment, and hopefully significantly decrease the morbidity and mortality of secondary fractures by using tools such as opportunistic imaging (X-ray and CT) that are already ubiquitous within an average clinic day.

## 2. Opportunistic Imaging

### 2.1. X-ray and Primary Fracture Prevention

While the current gold standard of osteoporosis diagnosis is by a DEXA scan, obtaining such a scan often requires a separate appointment and can represent a significant hurdle to patients, delaying or even preventing an initial diagnosis of osteoporosis from being made. In a study by Feldstein et al., less than 12% of 3812 women over the age of 50 with a new index fracture had a pre-fracture osteoporosis diagnosis. Even after sustaining a fracture, minimal numbers of those patients were shown to have obtained a proper BMD measurement in the months after sustaining the injury, with transportation issues being one of the cited barriers to care [13]. Additionally, it was found that only 46.4% of women in the study had been managed by recommended clinical osteoporosis guidelines (BMD measurement and/or initiation of medication within 6 months of the fracture) [13]. This study highlights the gaps in osteoporosis management in both pre-fracture and post-fracture situations, emphasizing the need for more efficient diagnostic and interventional strategies.

The use of opportunistic imaging to diagnose osteoporosis has been gaining attention as it represents a pre-existing resource that does not require any additional cost, medical appointments, tests, or procedures. Within our own multi-specialty practice, which sees almost 100,000 distinct patients per year, there are over 13,000 hand and wrist X-rays performed that can be used to analyze patients’ bone health. The general benefits of X-ray as an imaging modality are that it is low-cost, has low radiation, and is often obtained for reasons other than a distal radius fracture, therefore it can be used for the primary prevention of osteoporotic fractures.

Studies have shown that the use of a true PA wrist or hand radiograph can be used as a surrogate for DEXA scan to estimate BMD [14,15]. Without the need for extra software or complex analysis, two simple measurements of the mid-diaphysis of the second metacarpal (2MCP) can be used to screen for osteoporosis. Schreiber et al. conducted a study with 200 patients aged 20–89, and found that a 2CMP score significantly correlated with hip BMD and t-scores. The cortical percentage, as measured by the total width of the metacarpal minus the width of the intramedullary canal, multiplied by 100, significantly correlated with patients’ BMD measurements completed within one year of X-ray completion. It was found that a second metacarpal cortical percentage of <50% had 100% sensitivity and 91% specificity for distinguishing between osteoporotic patients and those with normal bone quality [15]. Burton et al. conducted a retrospective cohort study with 188 patients aged 49–90, comparing 2MCP from radiographs to femoral neck t-scores from DEXA scans [14]. Schreiber et al.’s study was verified via the multicenter study by Burton et al. which showed a 2MCP of <50% having 100% sensitivity and specificity.

O’Mara et al. also explored this concept in a study involving 43 female participants, both prospectively and retrospectively. They found that 2MCP measurements were strongly correlated with the BMD of the distal forearm. Additionally, they demonstrated that the 2MCP measurement could distinguish between osteopenia and osteoporosis with high sensitivity and specificity, even outperforming femoral neck t-scores [16]. This study provided detailed ROC curves showing optimal 2MCP cutoff values for diagnosing osteoporosis at 48.3% with a sensitivity of 80% [16]. This study further supports previous studies and highlights the significance of integrating 2MCP measurements into clinical practice. Building on this, Patel et al. conducted a retrospective study involving 206 patients who had both a hand radiograph and a DEXA scan within one year of each other. They found a statistically significant relationship between t-scores and a second metacarpal cortical index score (*p* < 0.001) [17].

Collectively, these studies show the potential of using routine hand X-rays to diagnose osteoporosis. This is particularly valuable as it uses existing imaging resources, is financially effective, and involves minimal radiation, making it an efficacious and accessible option for primary fracture prevention in osteoporotic patients.

### 2.2. Computed Tomography and Secondary Fracture Prevention

Computed tomography (CT) images are also a useful resource for evaluating bone quality. The Hounsfield Unit (HU), or the CT unit, is a measurement of radiodensity. The denser a tissue is, the greater the beam absorption, conversely, the less dense a tissue is, the less beam is absorbed. For context, air is defined as −1000 HU, distilled water is defined as 0 HU, and metals such as steel can reach over 3000 HU [18]. A typical range of trabecular to cortical bone can measure anywhere from 300 to 3000 HU [19]. Since this is a relative scale, it is important to note that variables in CT scanners and their settings can cause differences in HU measurements and should be considered when using HUs clinically [20].

Because CT scans involve a much higher level of radiation exposure to patients than either conventional X-rays or DEXA scans, they are not a good option for primary osteoporosis screening, however, if already completed for other indications, they can be used opportunistically as a data resource at no extra cost. The precedent for this has been well established in spine literature, using CT scans of the chest, abdomen, and pelvis completed in the emergency department to evaluate the bone quality of vertebral bodies [20]. Within the context of an upper extremity surgeon’s clinic, patients who have sustained a distal radius fracture may undergo CT scans of the wrist for surgical planning purposes. This represents a chance for CT scans to be used in diagnosing osteoporosis, leading to appropriate initiation of treatment and secondary fracture prevention. This is a critical time to catch undiagnosed osteoporosis as these patients are at high risk of more serious and highly morbid fractures in the future.

Multiple sites of measurement have been studied, including the distal radius, ulnar head, and capitate, all of which have shown promising results [21]. Schreiber et al. studied the CT scans of 50 patients who had suffered a distal radius fracture, and compared them to the CT scans of 50 patients who underwent CT scans for other indications. There were 25 males and 25 females in each age group, and the average age in the fracture cohort was 44.5, while the average age in the control cohort was 44.7. They measured the trabecular bone HU values at the distal radius metaphysis, ulnar head, and capitate, all of which showed a statistically significant difference between cohorts. While they made several measurements in serial cuts of each location, they found that there was no difference between the cuts at a single location, meaning a single measurement at a single location is likely sufficient for a regional BMD evaluation. After optimizing their data for sensitivity and specificity, Schreiber et al. suggested a cut-off of 246 HU for males and 218 HU for females, as measured at the distal radius metaphysis. Patients with measurements below these thresholds would alert a provider to further evaluate for the presence of osteoporosis.

Johnson et al. studied 45 female patients with a mean age of 66.9 who had suffered a distal radius fracture. They measured the HU value at the capitate and compared it to the BMD and t-scores of the femoral neck, hip, and lumbar spine measured via DEXA scan within 12 months of the distal radius fracture [22]. They found that the capitate HU value was positively correlated with all measurements, and a cut-off of 307 HU would provide a sensitivity of 86% and specificity of 94% for detecting osteoporosis. Their results validate the notion that clinical CT scans can have a role in detecting and diagnosing osteoporosis. In addition to finding correlations between DEXA results and HU values on wrist CT, HU measurements have also been found to reliably identify patients who are at higher risk of future fragility fractures [23]. With a minimum five-year follow-up, Dworak et al. examined 157 patients’ wrist CT scans (83 female and 74 male) for the HU value of the distal ulna and their subsequent medical records for fragility fracture of the hip, spine, rib, or proximal humerus. The patients’ average age in the fragility fracture group was 59.7, and the average age in the non-fracture group was 54.9. They found that the cohort of patients who eventually sustained a fragility fracture had a significantly lower HU value than their counterparts. They established an odd’s ratio of 7.4 for future fractures in patients with low HU values [23]. This study demonstrates the predictive value of distal ulnar HU measurements for future fragility fractures. This could assist patients who have already suffered a distal radius fracture and are singled-out to undergo CT, but would also allow providers to catch patients with low BMD undergoing CT for non-fracture indications, allowing for the initiation of treatment or preventative strategies.

## 3. Discussion

The use of plain radiographs as tools to quantitatively diagnose osteoporosis is not a new concept. Prior to the advent of DEXA scans, measurements such as cortical thickness in the femur were used [24]. With the development of DEXA, conventional radiographs were largely abandoned as a standard method of diagnosis, however, some assessments remain X-ray-based, such as the spinal fracture index, which is used to grade the extent of vertebral fracture based on height reduction and morphologic change over time, which is useful for serial-based examinations [20]. New technology has also incorporated machine-learning algorithms implemented with software tools to obtain objective measurements based on conventional radiographs, eliminating interobserver variability [25]. However, this presents financial and accessibility issues, as not all providers will own these computer programs, whereas all current providers have access to plain radiographs.

Incorporating simple measurements such as the 2MCP into the standard evaluation and documentation of hand and wrist radiographs in patients over the age of 50, particularly female patients, could represent a powerful tool for upper extremity surgeons to combat osteoporosis and contribute to primary fracture prevention. Even if these measurements are used for the singular purpose of patient education and initiating the conversation regarding osteoporosis and its risks, it would represent an improvement upon the current state of osteoporosis treatment as many patients cite a lack of understanding or knowledge as a barrier to care [13].

While the current literature is very promising, there is no currently agreed-upon threshold for HU value at any location in the upper extremity that is diagnostic of osteoporosis. Further studies with larger patient cohorts would be useful in establishing a defined diagnostic threshold. Additionally, the field must keep in mind that HU values are measured on a relative scale and can be affected by the calibration of a single CT scanner. Despite these current barriers, using CT scans that are already available to obtain HU values at either the distal radius, ulna, or capitate seems to represent a useful barometer of bone health that requires little time to evaluate, no additional risk to the patient, and the possibility of preventing highly morbid injuries associated with undiagnosed osteoporosis.

### 3.1. Initiating Treatment of Osteoporosis

Currently, osteoporosis is underdiagnosed and undertreated, with only 25% of patients who sustain distal radius fractures undergoing evaluation of osteoporosis, and similar rates of patients who qualify for medical intervention under current guidelines being actively treated [26,27]. Orthopedic surgeons have proven to be an important influence in moving the needle on this prominent issue, with some studies showing an increase in the rate of DEXA scans ordered and completed, and treatment option education by primary care physicians rising to over 70% when initiated by an orthopedic surgeon [27]. Simple discussion, education, and referrals cannot be overemphasized as important treatment tools, as patients cite a lack of knowledge as a primary barrier to pursuing treatment [13]. Formalized education programs and referral pathways like fracture liaison services (FLS) have been shown to also significantly improve diagnosis and treatment of osteoporosis [6]. Incorporation of osteoporosis education and treatment into active hip fracture pathways, often run by a multi-disciplinary team including endocrinologists and geriatricians, is another option if the development of an FLS is too onerous [28]. While these formalized programs are beneficial, a singular provider, particularly one whose practice is primarily outpatients, may feel unable to initiate such an undertaking. However, these providers should continue to initiate osteoporosis treatment, as meta-analysis studies have shown equivalent efficacy for increasing evaluation and treatment of osteoporosis when initiated by both orthopedic surgeons and an FLS [12].

Going one step beyond education and appropriate referrals is initiating medical treatment of osteoporosis. Initiation and management of pharmacotherapy can be intimidating for both providers and patients. Many barriers stand in the way between the prescription and successful adherence to a recommended medication. Patients’ fears of side effects and cost play a role in non-adherence to prescriptions [13]. For orthopedic surgeons, cost and side effects also play a role, but, in addition, the inability to provide adequate follow-up for monitoring once prescription medication has been initiated also does [29]. Insurance can also represent a barrier. For example, Medicare will cover the cost of bisphosphonates if there is a fragility fracture of the spine or hip, or if there is a fragility fracture of the distal forearm and the presence of diagnostic DEXA results [30]. For upper extremity surgeons, this would mean that, even if a provider felt comfortable initiating bisphosphonate therapy despite fears of cost, side effects, or follow-up, it may not be covered by insurance until a DEXA scan is performed. These are often co-ordinated by the patient’s primary care physician and may not be completed during the indicated follow-up period with the orthopedic surgeon.

While prescribing medication such as bisphosphonates, hormones, or biologics may not be feasible for orthopedic surgeons due to these barriers, supplementation of vitamin D and calcium is a more attainable goal. These supplements have far fewer side effects and contra-indications, and can be found over the counter, in addition to through formal prescription [27]. If patients are averse to taking any oral therapy, lifestyle changes to increase dietary calcium and vitamin D can be encouraged. The goal of supplementation is to attain a vitamin D level of 30–40 ng/mL, and the recommended daily dose for patients older than 50 who have sustained a fragility fracture is 1200 mg of calcium and 1000 IU of vitamin D [27]. Although it seems small, this intervention has been proven to significantly reduce fragility fractures and increase BMD [31].

### 3.2. Clinical Implications and Future Directions

Orthopedic surgeons routinely obtain imaging at the majority of clinic visits for both general evaluation of the bony anatomy and to correlate with specific patient complaints. X-rays are commonly obtained either before or during the clinic visit, whereas CT scans are more often obtained after the clinic visit. Providers then will either call the patient to discuss the results or set up another clinic visit to go over the CT scan. By incorporating simple measurements such as second metacarpal cortical percentage from X-rays and Hounsfield Units from CT scans into these routine evaluations, surgeons can play a crucial role in preventing secondary fractures. Utilizing these extra measurements would hardly affect a provider’s usual workflow as they are quick to make, adding less than a minute to the standard evaluation of the imaging. No formal training would be required as all radiology software have these basic functionalities, rather, orthopedic surgeons simply need to be educated on the benefits of implementing these measurements.

The cost effectiveness of opportunistic imaging lies in its ability to utilize existing radiographic resources. Integrating osteoporosis screening into routine orthopedic evaluations such as a wrist X-ray can combine existing imaging with early osteoporosis detection at no additional cost. Moreover, the early detection facilitated by opportunistic imaging can reduce the incidence of costly osteoporotic fractures, like hip fractures which are associated with massive healthcare expenses such as surgery, hospitalization, and physical therapy services [32,33]. Treating a hip-fracture patient costs about three times more than caring for a patient without such a fracture, and is estimated to be over USD 7300 in excess costs [33]. Opportunistic imaging presents a financially viable strategy to prevent such expenses, especially in areas of economic instability. Future studies could examine the relationship between costs associated with fragility fractures both before and after opportunistic imaging is implemented as a resource for osteoporosis screening.

Further research is needed to standardize the diagnostic thresholds for Hounsfield Units and other radiographic measurements in the diagnosis of osteoporosis. Future longitudinal studies should explore the long-term outcomes of patients diagnosed with osteoporosis through opportunistic imaging and the early intervention on fracture rates and overall patient health. Development of universally accepted guidelines for using opportunistic imaging in osteoporosis screening will enhance the consistency and reliability of these methods across different clinical settings. Furthermore, implementing educational programs for upper extremity surgeons and other involved healthcare providers on the use of opportunistic imaging can increase the awareness and adoption of these techniques.

## 4. Conclusions

In summary, osteoporosis provides an increasing burden on the healthcare system, and even though there are well-established methods for diagnosis and management of osteoporosis, the widespread implementation on high-risk populations with fragility fractures is still lacking. Presently, only 25% of patients who have sustained a fragility fracture are evaluated for osteoporosis. Part of the barrier to care for these patients is undergoing testing and treatment outside orthopedic offices where fragility fractures are first diagnosed. Current tests, such as X-rays and CT scans routinely used in the orthopedic office, can be used to screen higher-risk populations. As previously established, when osteoporotic workups are initiated by orthopedic surgeons, the evaluation rates increase to 70%. Incorporating simple methods such as the second metacarpal cortical percentage (2MCP) being less than 50% on X-rays can help the sensitivity and specificity of diagnosing osteoporosis by nearly 100%. If CT scans of the distal radius have been utilized, evaluating the Hounsfield units at the distal radius metaphysis or the capitate can also be used to screen for osteoporosis. Identifying high-risk patients who have sustained fragility fractures and have a substantial likelihood of osteoporosis, given the radiographic indices described above, is likely to have a positive effect on their overall medical treatment. While initiating osteoporosis treatment with bisphosphonates, hormones, or biologics may still require referral to a medical specialist, routine recommendations for daily supplementation in patients older than 50 with 1200 mg of calcium and 1000 international units of vitamin D can be widely implemented.

To be effective in addressing osteoporosis, it is crucial for healthcare providers, healthcare systems, and even policymakers to be proactive. Along with incorporating opportunistic imaging into routine orthopedic assessments, upper extremity surgeons should engage in collaboration with other disciplines such as primary care physicians, endocrinologists, and geriatrics to ensure comprehensive osteoporosis management. Healthcare systems must continue to develop and implement clinical guidelines that utilize opportunistic imaging for osteoporosis screening in high risk patients, while also providing ongoing education around these strategies. Finally, we advocate for supporting further research to validate and refine opportunistic imaging techniques to ensure consistency and effectiveness across all clinical settings.

## Data Availability

No new data were created or analyzed in this study. Data sharing is not applicable to this article.
